# Qualitative and quantitative analysis of reduced bed position acquisition time on FDG PET image quality

**DOI:** 10.1097/MNM.0000000000001957

**Published:** 2025-01-21

**Authors:** Michael Ting, Garry McDermott, Amir Zarei, Chirag Patel, Fahmid U. Chowdhury, Andreia Rodrigues, Andrew F. Scarsbrook

**Affiliations:** a Department of Nuclear Medicine,; b Department of Medical Physics & Engineering, Leeds Teaching Hospitals NHS Trust,; c Leeds PET Centre, Alliance Medical Ltd, St James’s University Hospital and; d Faculty of Medicine & Health, Leeds Institute of Medical Research, University of Leeds, Leeds, UK

**Keywords:** fluorodeoxyglucose, image reconstruction, improvement, PET-computed tomography, quality

## Abstract

The study aim was to evaluate whether reducing bed position acquisition time would result in significant detriment to image quality. Secondary aims were to compare effect of time of flight (TOF) and Q.Clear reconstructions and patient BMI on image quality. Fluorodeoxyglucose PET-CT performed in 30 patients on a new scanner at our institution between March and May 2024 was retrospectively evaluated. Four PET reconstructions were performed: (a) 1 min 45 s TOF, (b) 2 min TOF, (c) 1 min 45 s Q.Clear, and (d) 2 min Q.Clear. For qualitative analysis, four maximum intensity projection images were evaluated side-by-side using a five-point visual score (1 = non-diagnostic, 5 = excellent). For quantitative analysis, liver signal-to-noise ratio (SNR) was calculated. A statistically significant reduction in visual score occurred when reducing bed position time from 2 min to 1 min 45 s (mean TOF scores 0.24 reduction, *P* = 0.0002; mean Q.Clear scores 0.04 reduction, *P* = 0.02. There was also a statistically significant difference in liver SNR when reducing bed position time. Deterioration in image quality was minimised when bed position acquisition time was reduced if Q.Clear construction was utilized. This could facilitate increased scanning capacity without clinical detriment.

## Introduction

Fluorine-18 fluorodeoxyglucose (FDG) PET-computed tomography (PET-CT) is widely utilized in the diagnosis and monitoring of oncological, rheumatological, and immunological diseases [1–4]. In recent years, technological advances in PET reconstruction algorithms have improved image quality. Proprietary reconstructions by the main scanner manufacturers incorporate point source function into so-called high-definition algorithms. GE Healthcare have developed a Bayesian penalized likelihood reconstruction algorithm, referred to commercially as Q.Clear [5]. Q.Clear has been shown to improve contrast recovery and reduce background variability compared to traditional time of flight (TOF) reconstruction, resulting in improved signal-to-noise ratio (SNR) [6–9]. Rising demand for PET-CT scans has put pressure on nuclear medicine departments to increase scanning capacity. The improved image quality and lesion detectability afforded by Q.Clear might offer a potential solution by allowing a reduction in scan acquisition time. One group has recommended using an activity time product (administered activity × acquisition time) >6 to ensure good image quality and lesion detection rate [10].

The primary aim of this study was to evaluate whether reducing scan time per bed position could be performed without significant detriment to image quality at our institution. Secondary aims were to compare the quality of Q.Clear and TOF reconstructions and the confounding effect of patient body mass index (BMI) on image quality to inform optimization of scanner workflow following installation of a new digital PET-CT system.

## Methods

### Patent selection

Formal ethics committee approval and informed written confirmation was waived for this study, which was considered by the institutional review board to represent evaluation of a routine clinical service. Imaging data from 30 individual patients undergoing FDG PET-CT studies on a newly installed PET-CT scanner at our institution were selected retrospectively between March 2024 and May 2024. Patients were chosen to represent a real-world range of body weights.

### Image acquisition and analysis

PET-CT imaging was performed on a GE Healthcare MI Discovery Generation 2 digital scanner (GE Healthcare Technologies, Inc., Chicago, Illinois, USA). Dose of FDG injection was calculated as 3.5 MBq × patient body weight (kg), with a maximum injected activity of 400 MBq. The FDG uptake period was between 55 and 75 min, as per European Association of Nuclear Medicine guidelines [11]. Length of coverage per bed position was 25 cm, which includes a 35% overlap between adjacent bed positions. Retrospectively, four image reconstructions were performed, which were (a) 1 min 45 s TOF, (b) 2 min TOF, (c) 1 min 45 s Q.Clear (beta 700), and (d) 2 min Q.Clear (beta 600).

**Fig. 1:**
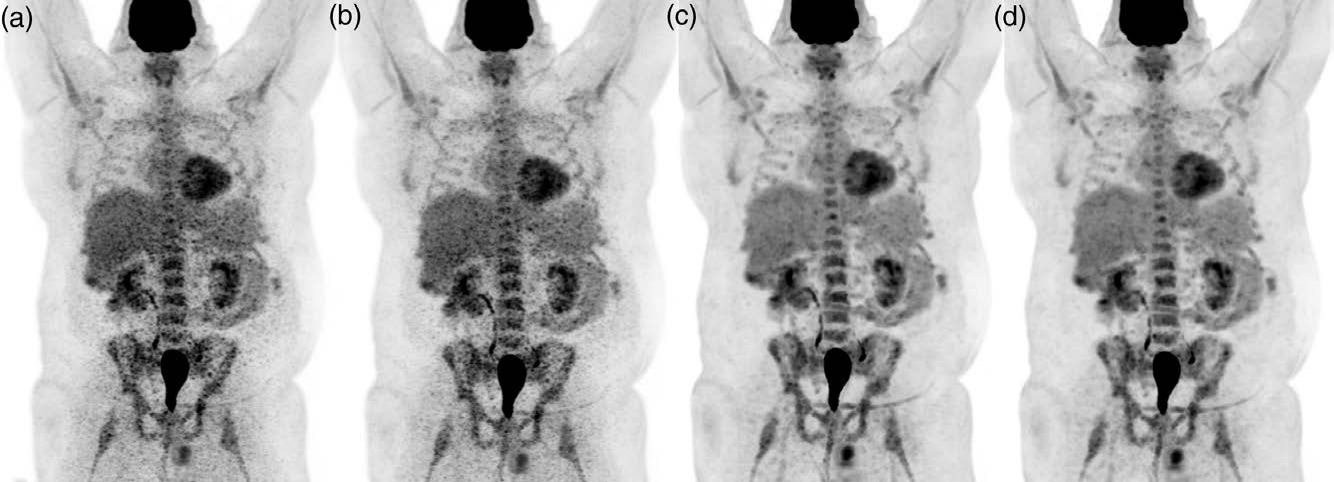
Maximum intensity projections of (a) 1 min 45 s time of flight, (b) 2 min time of flight, (c) 1 min 45 s Q.Clear, and (d) 2 min Q.Clear PET reconstructions in an example patient.

Qualitative analysis was performed independently by three experienced consultant radiologists and one radiology registrar. Using GE Advantage Windows Version 4.5 (GE Healthcare Technologies, Inc., Chicago, Illinois, USA), the four maximum intensity projection images (one from each reconstruction) were evaluated side-by-side facilitating direct comparison and visual scoring (Fig. 1). Visual PET image quality was assessed using a five-point ordinal scale (5, excellent; 4, good; 3, moderate/acceptable; 2, suboptimal/poor; 1, nondiagnostic). A mean visual score was calculated for each reconstruction encompassing the scores of all readers.

Quantitative analysis was performed by an experienced medical physicist with expertise in PET imaging using liver SNR. Liver SNR was determined by placing a 5 cm diameter volume of interest on the liver and extracting mean and SD values (avoiding blood vessels and tissue boundaries) [12]. Liver SNR was defined as the ratio of mean to SD of the activity concentration in the volume of interest.

### Statistical analysis

The mean and SD of the visual scores for each reconstruction were calculated using Microsoft Excel Version 16.89 (Microsoft Corporation, Redmond, Washington, USA). Comparative graphs of visual scores against BMI were plotted using the same software. To assess for statistically significant difference, paired samples *t*-test was calculated with the same software. A *P*-value of <0.05 was taken to be statistically significant.

Graph of liver SNR against weight was plotted using Microsoft Excel Version 16.89.

## Results

The sample size was 30 patients (9 female, 21 male). The age range was from 23 to 86 years (mean age 62). The weight (kg) range was from 40 to 205 kg (mean 94 kg, SD 34). The BMI range was from 14.7 to 59.9 (average BMI 30.8, SD 8.8).

Table 1 summarizes the mean and SD of the visual scores for each of the four reconstructions. There was a statistically significant reduction in visual score when reducing bed time from 2 min to 1 min 45 s per bed position. The mean Q.Clear scores were reduced by 0.04 (*P* = 0.02), whereas mean TOF scores were reduced by 0.24 (*P* = 0.0002).

**Table 1:** Mean and SD of visual scores for each image reconstruction type

Type of image reconstruction	Mean visual score	SD
Q.Clear 2 min	4.93	0.21
Q.Clear 1.75 min	4.89	0.26
TOF 2 min	4.17	0.44
TOF 1.75 min	3.93	0.52

TOF, time of flight.

**Fig. 2:**
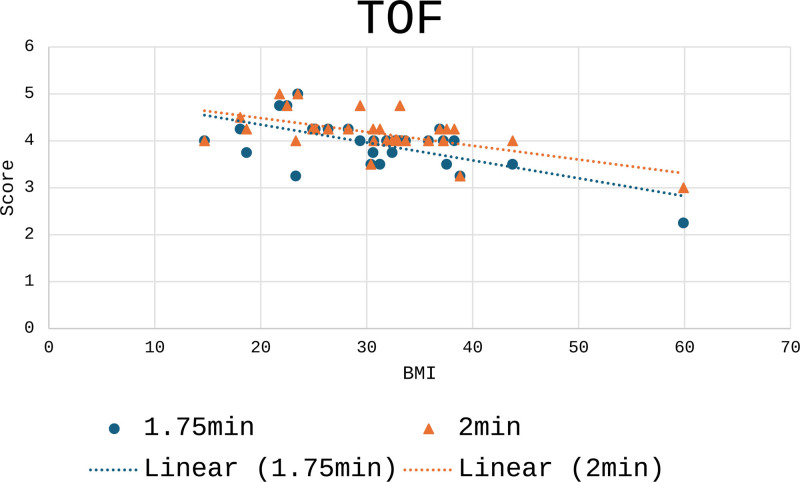
Graph of visual score against BMI in TOF reconstructions. TOF, time of flight.

Figure 2 illustrates the spread of visual scores for 1 min 45 s TOF and 2 min TOF across a range of BMIs. Overall, the 2 min TOF images were visually rated higher than the 1 min 45 s TOF images (*P* = 0.000261).

**Fig. 3:**
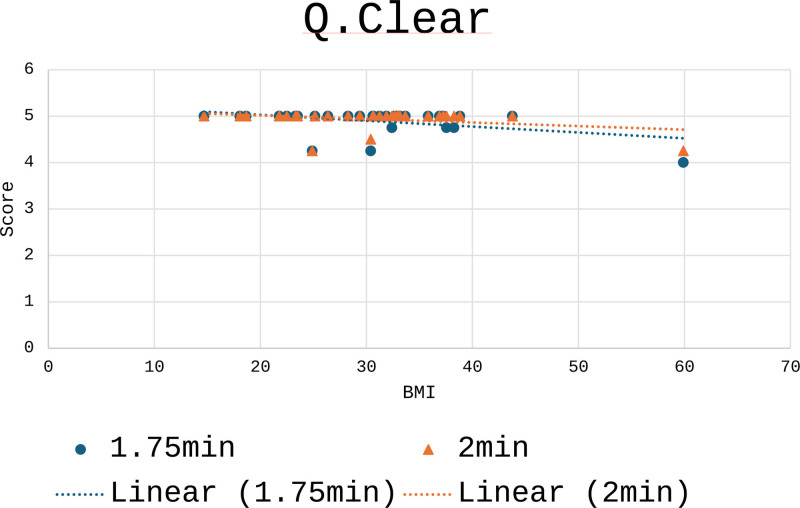
Graph of visual score against BMI in Q.Clear reconstructions.

Figure 3 shows the spread of visual scores for 1 min 45 s Q.Clear and 2 min Q.Clear across a range of BMI. Overall, the 2 min Q.Clear images were visually rated higher than the 1 min 45 s Q.Clear images; however, the difference was fairly minimal but still statistically significant (*P* = 0.022).

**Fig. 4:**
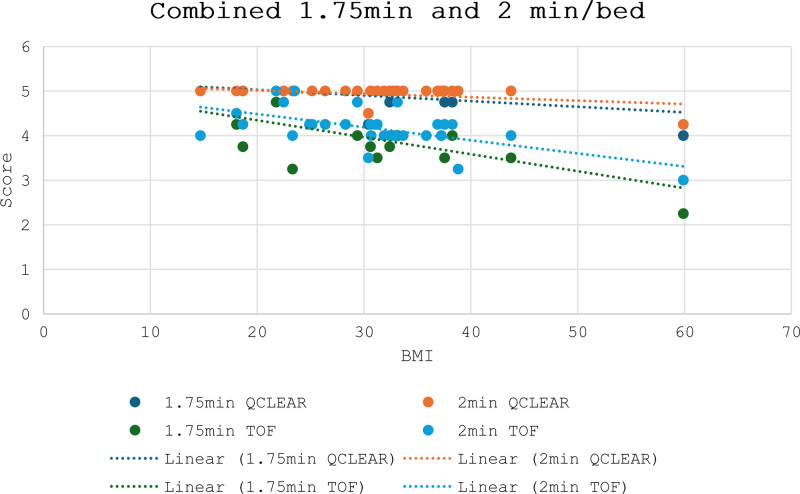
Spread of visual scores against BMI for all four reconstructions.

Figure 4 shows the spread of visual scores for all four reconstructions across a range of BMIs. Q.Clear images were shown to be visually rated higher than the TOF images. The TOF images showed a steeper gradient of decline of visual scores with increasing BMI.

**Fig. 5:**
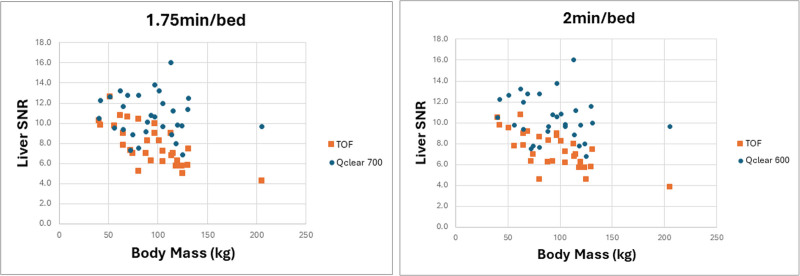
Graph of liver signal-to-noise ratio (SNR) against body mass at (i) 1 min 45 s and (ii) 2 min.

Figure 5 shows the spread of quantitative scores at 1 min 45 s and 2 min bed time, respectively. There was a statistically significant difference in liver SNR when reducing bed time from 2 min to 1 min 45 s per bed position, with a greater difference observed in the TOF reconstruction compared to Q.Clear (*P* = 0.03 for Q.Clear and *P* = 0.00007 for TOF).

## Discussion

There was a statistically significant reduction in visual scores when reducing bed time from 2 min to 1 min 45 s per bed position. The mean Q.Clear scores were reduced by 0.04 (*P* = 0.02), whereas mean TOF scores were reduced by 0.24 (*P* = 0.0002). The 15 s reduction in bed time resulted in a greater reduction in TOF visual scores, compared to Q.Clear.

At the same bed time per position, Q.Clear images were visually rated higher than TOF. 2 min Q.Clear was the best visually rated, followed by 1 min 45 s Q.Clear, 2 min TOF, and lastly, 1 min 45 s TOF.

There was a much steeper decline in TOF visual score with increasing BMI. With increasing BMI, the discrepancy between Q.Clear and TOF visual scores increased.

From a quantitative perspective, there was a statistically significant difference in liver SNR for 1 min 45 s/bed and 2 min/bed, with a greater difference observed in the TOF reconstruction compared to Q.Clear. There is a marked decline in liver SNR with increasing body mass for TOF reconstructions, which indicates the protocol does not adequately compensate for increasing attenuation and scatter in higher body mass patients.

Previous optimization work performed by the department has shown a liver SNR > 9 was associated with good/excellent image quality scores and liver SNR < 5 was associated with poor/nondiagnostic image quality scores [12]. For this study, the SNR for the TOF images were mostly in the moderate (5–9) range, whereas the SNR for the Q.Clear images were mostly in the good (>9) range.

## Limitations

There was a small sample size for this study (*n* = 30). In addition, not all weight ranges were equally represented. There was one extreme outlier with a BMI of 59.9, while the second highest BMI was 43.8.

### Conclusion

Although there was a statistically significant reduction in visual scores when reducing acquisition time for both reconstructions, TOF images were more susceptible to image quality reduction compared to Q.Clear reconstructions. TOF reconstructions also showed a greater reduction in liver SNR compared to Q.Clear reconstructions. The minimal deterioration in Q.Clear images may provide a feasible solution to increase scanning capacity of nuclear medicine departments.

There was a general deterioration in image quality with increasing body mass. Nonlinear dosing or scan duration protocols may be required to improve image quality at higher weight ranges.

## Acknowledgements

### Conflicts of interest

There are no conflicts of interest.
